# Detection of Micrometer-Sized Virus Aerosols by Using a Real-Time Bioaerosol Monitoring System

**DOI:** 10.3390/bios14010027

**Published:** 2024-01-02

**Authors:** Hyunsoo Seo, Young-Su Jeong, Jaekyung Bae, Kibong Choi, Moon-Hyeong Seo

**Affiliations:** 1 Chem-Bio Technology Center, Advanced Defense Science and Technology Research Institute, Agency for Defense Development, Daejeon 34186, Republic of Korea; skhsjwjw@add.re.kr (H.S.); baejkj123@add.re.kr (J.B.); kibongchoi@add.re.kr (K.C.); 2 Natural Product Research Center, Korea Institute of Science and Technology (KIST), Gangneung 25451, Republic of Korea; mhseo@kist.re.kr

**Keywords:** biological weapons, bio-aerosol, virus aerosol, M13 bacteriophage, inkjet aerosol generator

## Abstract

This study investigates a real-time handheld bioaerosol monitoring system for the detection of biological particles using UV-LED and light-induced fluorescence technology. Biological particles produce both scattering and fluorescence signals simultaneously, which can help distinguish them from general particles. The detected scattering, fluorescence, and simultaneous signals are then converted into photon signals and categorized based on predetermined criteria. A reliable biological particle generator was required to validate the performance of the system. This study explores the use of an M13 bacteriophage as a virus simulant of biological agents and employs a customized inkjet aerosol generator to produce M13 bacteriophage aerosols of a specific size by controlling the concentration of M13. We confirmed that micro-sized, narrowly dispersed M13 aerosols were efficiently generated. Additionally, we confirmed the performance of this real-time handheld bioaerosol monitoring system by detecting viruses.

## 1. Introduction

Biological weapons can cause mass casualties even at very low concentrations [1]. The biological agents used in these weapons reproduce and incubate themselves after infecting the human body and can cause severe damage to an unspecified number of people in a short duration because of their rapid spread after infection [2]. In addition, because they are not visible to the naked eye and have no smell or taste, they are difficult to detect. Failure to detect them early can seriously threaten public health and safety [3]. Due to the risk of potential damage from unknown novel infectious diseases, such as COVID-19, interest in bioaerosols (viruses, bacteria, and molds) is increasing [4]. Real-time detection equipment is required to quickly determine whether a biological weapon attack has occurred. The Agency for Defense Development has developed a real-time handheld bioaerosol monitoring system (RT-HBMS) via a technology that detects biological particles in real time using a 280 nm UV-LED. This device is based on light-induced fluorescence technology, where the particles suspended in air are adsorbed, passed through a nozzle, and irradiated with ultraviolet light at the end of the nozzle [5]. When light is irradiated onto a biological particle, scattering and fluorescence occur [6]. Measurement of the scattering and fluorescence signals with separate detectors has shown that general particles produce only a scattering signal, whereas biological particles produce both scattering and fluorescence signals simultaneously, which can help distinguish between the two. In this optical setup, the introduction of fine particles into the flow path within the optical chamber triggers their interaction with a light source, resulting in the generation of optical signals primarily concentrated at the central focus. These signals are captured through a series of techniques, including immediate collection at the second focus or reflection by an aspherical mirror within the optical cell followed by secondary collection. In certain scenarios, they are detecting using reflection by an internal spherical mirror, a return journey through the central focus, subsequent reflection by the aspherical mirror, and final collection at the second focus. These designed configurations aim to significantly boost sensitivity by optimizing the collection of weakly scattered light and fluorescence. The collected light signals, taken from outside the optical chamber, are then directed to a detector for interaction and analysis. Any residual light is effectively eliminated using a beam dump strategically positioned opposite the excitation sources [7]. A comprehensive process is used for optical signal processing and particle size classification within a system featuring a transmission wavelength range of 255 to 290 nm and a fluorescence-blocking filter. This process begins with the acquisition of scattering and fluorescence signals through a photomultiplier tube (PMT), which are subsequently converted into digital signals for utilization in the detection algorithm. The signal processing method involves multiple stages, including signal amplification through the conversion of analog optical signals into voltages, followed by amplification using an analog front-end amplifier. The resulting amplified signals, denoted as V_FT_ (voltage-of-fluctuation signals), are further converted into digital form by comparing them to a predetermined threshold voltage, V_THD_, and then counted based on varying digital pulse widths corresponding to signal intensities. A digital complex programmable logic device (CPLD) serves as an intermediate step, sampling the pulse width signals at 1 MHz and forwarding them to a microcontroller unit (MCU) if a logical value of “1” is detected. The MCU ultimately receives and processes the counted scattering and fluorescence signals, classifying them into “small”, “medium”, and “large” categories based on predetermined particle size thresholds, originally established within the range from 0.84 to 11 µm in an experimental study, aimed at detecting biological weapon particle sizes from 1 to 10 µm. This well-structured algorithm ensures efficient and precise particle size determination within the specified range [8,9]. Biological weapons have a particle size ranging from approximately 1 to 10 µm, and a blocking threshold is set for particle sizes within this range. This threshold has been determined experimentally in previous research and adjusted to block small (0.84–2 µm), medium (2–5 µm), and large (5–11 µm) particles. This particle size classification was determined by taking a common logarithm of the data to ensure normality, ranging from 1 µm to 10 µm. Using the common logarithm, the median value is 3 µm, and 2–10 µm is considered the size range for the intermediate particles, with a median value of 3 µm [10,11,12].

Developing a biological particle detection system and validating its performance requires a reliable biological particle generator. There are few cases where viruses themselves are generated as monodisperse particles in the micrometer size range [13]. 1. Among the existing techniques for generating known biological particles, the collison nebulizer, which utilizes the twin-fluid atomization method, is one. This method, while generating particles, causes damage to biological particles due to the use of fluid force and recirculation, resulting in polydisperse particle generation [14]. 2. Sparging liquid aerosol generators (SLAG) generate particles through bubble bursting, resulting in particle damage due to fluid force, producing polydisperse particles [15]. 3. Commercial nebulizers such as the Sidestream nebulizer and PariLC Sprint use the venturi principle to generate particles, producing polydisperse particles as well [16]. 4. The flow focusing monodisperse aerosol generator (FMAG) allows particle size control through pressure and flow rate adjustment but requires extensive experimentation to find the optimal conditions [17]. 5. The spinning top aerosol generators (STAG) employ a disc for particle generation, but device maintenance and cleaning after experiments are significantly time-consuming [18]. 6. Electrospray ionization is suitable for generating very small particles, but these particles are smaller than those within the target size range of 0.84–10 µm, which is relevant for biological agents [19]. 7. The rotating brush generator (RBC) generates particles using scraping/mixer-style blades or impellers, resulting in polydisperse particle generation [20]. 8. The Vilnius aerosol generator, which forces powdered/dried material through a bed of heavy particles fluidized by air, also leads to polydisperse particle generation [21]. While there is a variety of biological particle generators available, there are hardly any that can generate particles within the size range of 0.84–10 µm as monodisperse particles. Therefore, the novelty of our research lies here, and to determine the detection efficiency of various biological particle detectors, we need to use an inkjet system to generate monodisperse particles. This is primarily carried out to assess efficiency at specific particle sizes. In particular, this particle generator allows for easy concentration adjustment, which is a unique feature. Normally, obtaining the desired particle size requires extensive experimentation, but with this particle generator, you can set one condition and only adjust the solution’s concentration to control particle size. Through previous research, we have confirmed that it is possible to generate specific-sized biological particles with a narrow dispersion range based on the concentration of biological samples without adjusting the operating conditions of the complex hardware. Techniques for controlling the size of bioaerosol particles with a narrow distribution using a custom-made inkjet aerosol generator (IJAG) under fixed operating conditions that can control the bioaerosol model concentrations have been described [22,23,24]. Given the increased interest in virus detection due to the COVID-19 pandemic, it is essential to investigate the possibility of generating micro-sized virus particles. In this study, we explored the use of an M13 bacteriophage in our experimental setup to effectively control the particle size by controlling the concentration of M13.

## 2. Materials and Methods

**Figure 1:**
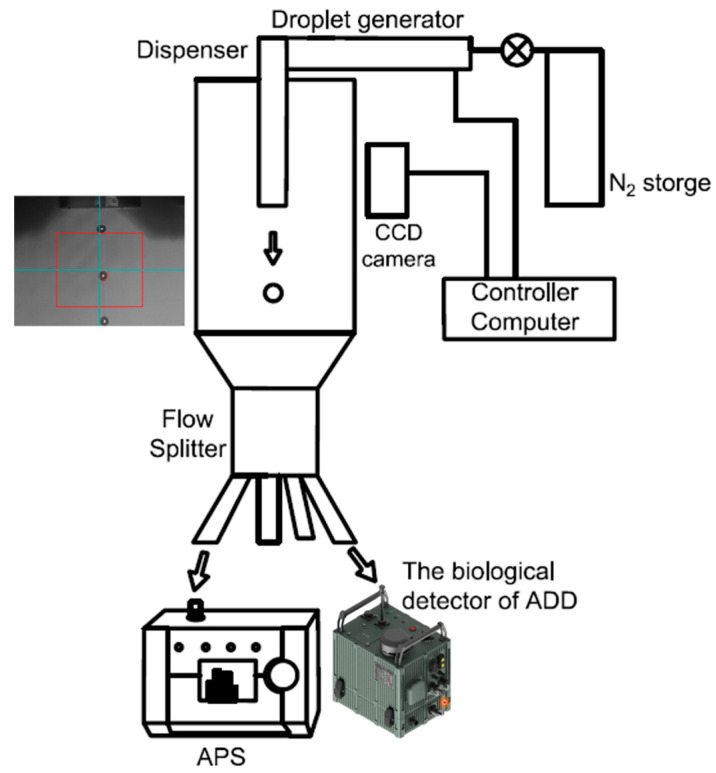
Design of the particle generator. Snapshot image of a droplet generated by a piezo-type inkjet nozzle in the customized inkjet aerosol generator (IJAG).

The M13 bacteriophage, used as a virus simulant of a biological agent, is a bacterial virus that contains mainly proteins and nucleic acids and has a circular single-stranded DNA (ssDNA) genome. It is typically introduced into Escherichia coli and amplified; the amplified viruses are secreted from the cell without killing the host *E. coli*. To produce M13 bacteriophage, *E. coli* TG1 cells containing a phagemid were grown in 30 mL of 2YT medium (16 g Bacto tryptone, 10 g Bacto yeast extract and 5 g NaCl in 1 L water) containing 100 μg/mL carbenicillin. The cells were infected with VCSM13 helper phage when the cells entered the log phase. After 30 min of incubation at 37 °C with shaking at 200 rpm, the cells were transferred to 1 L of 2YT medium containing 100 μg/mL carbenicillin and 25 μg/mL kanamycin and cultured for 16 h. The pelleted cells were removed by centrifugation at 12,000× *g* for 10 min, and the phage particles in the supernatant were precipitated by adding 200 mL of polyethylene glycol–NaCl, (20% PEG-8000 (*w*/*v*), 2.5 M NaCl) and centrifuging at 16,000× *g* at 4 °C for 20 min. The precipitated virus was washed with PBS buffer and then resuspended with PBT buffer (PBS with 0.05% Tween-20 and 0.5% BSA). Finally, to collected the desalted virus, the purified phages were desalted with a PD-10 desalting column (GE Healthcare, Uppsala, Sweden) using double-distilled water. The final concentration of the M13 bacteriophage was determined by measuring the absorbance at 268 nm. To generate an M13 bacteriophage containing droplets of a specific size, we employed a customized inkjet aerosol generator (IJAG) with a micro-droplet component (MD-K-130 Micro-droplet™, Microdrop Technologies Gmb, Norderstedt, Germany). The IJAG comprises an aerosol generator module, including a piezo-type inkjet nozzle and a reservoir, a control unit with a function generator and a charge-coupled device camera, flow splitter, a heater, and a drying module. The Micro-droplet™ allows for the control of the droplet size and frequency. After imaging the droplets using the CCD camera, their size can be measured. The IJAG also includes a drying module with a heater and a nitrogen gas supply, which can control the temperature of the gas to dry the liquid phase of the droplets and prepare a pure sample. The speeds of the droplets and pure samples could be controlled by adjusting the amount of nitrogen gas. The heater and flowmeter helped regulate the amount and velocity of the gas, which was supplied through a HEPA filter for cleanliness. Generally, the user can adjust the generation parameters of IJAG to achieve specific particle sizes, with the pulse width, voltage, and frequency affecting the droplet size and satellite drops [24]. The generated droplets could be observed in real-time using a droplet camera and analyzed using software based on their size. A revolutionary technique that adjusts particle size by changing only the concentration of biomaterial samples in fixed parameters has also been used [22]. To generate M13 bacteriophages, we used the following fixed conditions: a nozzle heating temperature of 140 °C, a driver voltage of 240 V, a pulse width of 100 µs, and a frequency of 1000 Hz. The particle size was changed by altering the concentration of M13 samples, with larger particles generated by diluting the sample to a lesser extent. To generate the M13 bacteriophage particles, spores with concentrations ranging from 2.4 × 10^8^ to 2.4 × 10^12^ plaque-forming units (PFUs) per mL in sterile water were adjusted using a piezo-type inkjet nozzle. Particles of various sizes were prepared by diluting half of the aforementioned solution. We generated narrowly dispersed particles with M13 bacteriophage-specific sizes, which were analyzed using an aerodynamic particle sizer spectrometer 3321 (TSI Inc., Shoreview, MN, USA), a standard analyzer for measuring the particle size distribution in real time [25]. IT500HR (version 1.23) software was used to analyze the SEM images captured using a JSM-7000F microscope (JEOL Ltd., Tokyo, Japan). The particles were collected using carbon tape to ensure the electrical grounding of the SEM specimen. The inkjet particle generator was connected to a four-split flow distributor (TSI Inc., Shoreview, MN, USA, Model 3708), which was then connected to a 1/4-inch Teflon tube of the same length. The device evenly divided the biological particles in four directions using one inlet with a 3/8-inch outer diameter and four outlets with a 1/4-inch outer diameter. The outlet area was made relatively wide to allow the particles to spread across the entire area inside the port, forming a uniformly distributed concentration. The device also has the advantage of selecting the number of ports to be used by blocking the unused ports [8]. During the 20 min collection period, one port was connected to the APS, one port to the RT-HBMS, and the other port to the carbon tape to collect particles of the same size. The customized IJAC can control the volume and frequency of the microdroplets and measure the size of individual microdroplets using a visualization camera (Figure 1). 

## 3. Results

**Figure 2:**
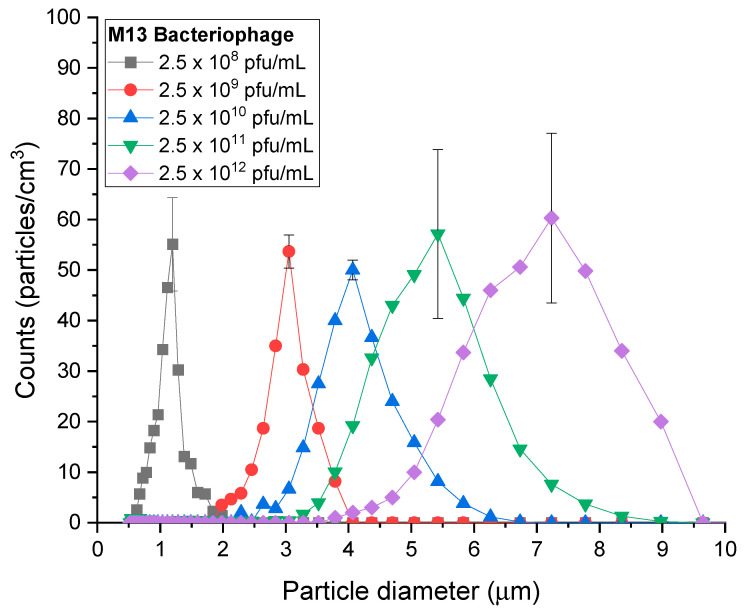
Size distribution of M13 bacteriophage aerosols.

As shown in Figure 2, the particles were successfully produced and quantified using APS. The generated M13 particle diameters were 1.197, 3.051, 4.068, 5.425, and 7.234 µm for each concentration. With the increase in particle size, the particle width increased, with concentrations of 55, 54, 50, 57, and 60 particles/cm^3^. The standard deviations for each particle were 9.24, 3.28, 1.96, 16.72, and 16.79 particles/cm^3^. With the increase in liquid density under fixed parameters, such as the density of the solution, a broad range of narrowly dispersed particles could be formed owing to the difference in the degree of evaporation under the thermal heater. 

**Figure 3:**
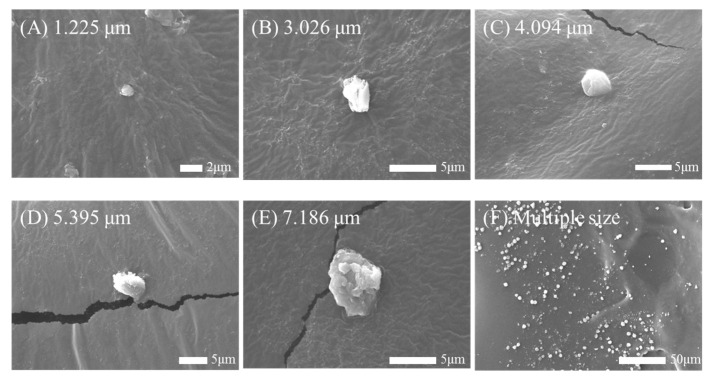
SEM images of M13 bacteriophage aerosol. (**A**) M13 of 1.225 µm, (**B**) M13 of 3.026 µm, (**C**) M13 of 4.094 µm, (**D**) M13 of 5.395 µm, (**E**) M13 of 7.186 µm, and (**F**) multiple sizes of M13, respectively.

The virus sample solution was transformed into particles of various sizes using a customized IJAC, as shown in Figure 3. Figure 3 shows the SEM results confirming the presence of M13 bacteriophage particles, obtained in sizes of 1, 3, 4, 5, and 7 µm, with each size obtained from M13 sample concentrations of 2.4 × 10^8^, 2.4 × 10^9^, 2.4 × 10^10^, 2.4 × 10^11^, and 2.4 × 10^12^ PFU/mL, respectively. The generated particles were confirmed using SEM and APS, and the size difference was within 3%. M13 dissolved in the solution was generated as a droplet. As it passed through the heater, the moisture was vaporized, and M13 was generated in an agglomerated state [24]. Figure 3F shows particles of various sizes produced by generating particles with sizes of 1, 3, 4, 5, and 7 µm each for 20 min and trapping them in one space. 

Finally, using narrowly dispersed M13 of 1, 3, 4, 5, and 7 µm size generated by IJAC, we confirmed that developing an RT-HBMS could detect viruses. The real size of the virus aerosol was confirmed through SEM and quantitative information via APS.

**Figure 4:**
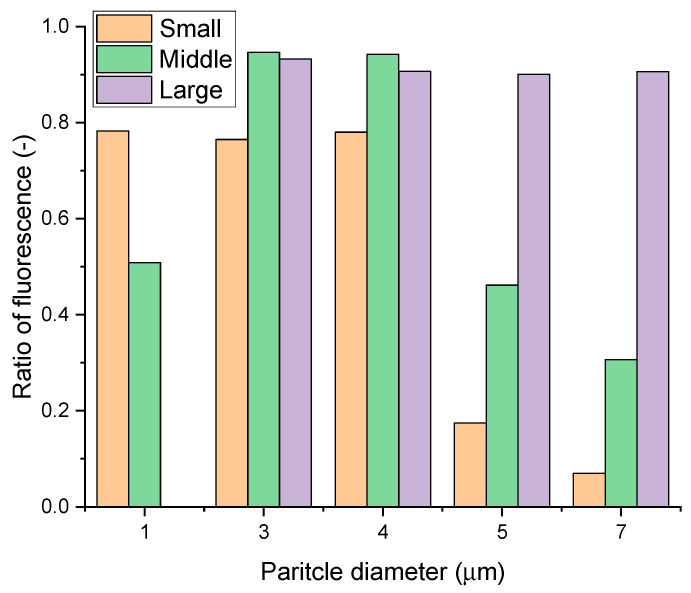
Ratio of fluorescence of the biological detector of ADD. The *x*-axis represents the average particle size generated. Virus particles generated with narrow dispersion at a specific size are classified into small, medium, and large according to the scattering intensity in the ADD biological particle detector and are indicated by orange, green, and purple bars, respectively. The *y*-axis represents the fluorescence ratio measured at that particle size (Table S1).

Narrowly dispersed particles of each size were generated to confirm the small, medium, and large fluorescence indices (fluorescence sum/scatter sum). This was carried out to determine the average distribution of the fluorescence index in various environments by obtaining the sum of the scattering particles and the sum of fluorescent particles measured as the most fundamental variables and by obtaining the proportion of the fluorescent particles in the total particles. Figure 4 displays the fluorescent index of particles at each particle size and, along with Figure 3, shows the particle size distribution. As the first basic parameter, the sums of fluorescence and scattering are calculated regardless of the magnitude, and the ratio of the sums of the total-particle fluorescence is obtained. From this, a fluorescence index with an average distribution in various environments is determined. Calculations are performed on data cumulated over 3 min, and, even if abnormal signals are identified, the time delay is maintained until the abnormal values are reflected in the average, which leads to accurate alarm generation. The fluorescence-index threshold is adjusted based on the sums of scattering and fluorescence. When the sum is high, the environment is determined to be polluted, and adjustments are made less sensitively. When the sum is low, the environment is determined to be clean, and the value can be adjusted more sensitively. To set different alarm thresholds based upon the sum, the weights of scattering and fluorescence distribution are predefined. For cases of biological weapons, the synthetic sizes are medium or large. Therefore, when the ratio is small, the environment is more susceptible to contamination. As the particle size increases, the size distribution becomes more expansive, making it easier to measure particle fluorescence.

Based on the size classification of the RT-HBMS, 1 µm corresponds to small particles, taking an index value of 0.78 for small particles, 0.51 to medium particles, and 0 to large particles. Based on the size classification of the RT-HBMS, 3 µm corresponds to a medium particle, taking an index value of 0.76 for small particles, 0.95 for medium particles, and 0.93 for large particles. Based on the size classification of the RT-HBMS, 4 µm corresponds to a medium particle, taking an index value of 0.78 for small particles, 0.94 for medium particles, and 0.91 for large particles. Based on the size classification of the RT-HBMS, 5 µm corresponds to a large particle, taking an index value of 0.17 for small particles, 0.46 for medium particles, and 0.90 for large particles. Based on the size classification of the RT-HBMS, 7 µm corresponds to a medium particle, taking an index value of 0.07 for small particles, 0.31 for medium particles, and 0.91 for large particles (Figure 4). For all sizes except 1 μm, it can be observed that over 90% of particles measured at the “large” standard of the small fluorescent measurement instrument exhibit fluorescence. Additionally, it can be confirmed that even for small particles with a size of 1 μm, the fluorescence ratio of the measured particles is 80%. In the RT-HBMS, a particle with a mode size of 1.197 µm in the APS has the highest fluorescence index for small particles, while a particle with a mode size of 7.234 µm in the APS has the highest fluorescence index for large particles. The fluorescence index appears in the boundary size of the particles even though it is not the corresponding particle. To account for optical and electrical noises, the particle size region was set to overlap when the particle judgment standard was established. 

The scattered light and fluorescence weights were added to establish the final alarm reference variable. To prevent the polluted environment value from affecting the reference variable during abnormal symptoms, a confidence interval was set based on the standard deviation of the effective biological particle, assuming a normal distribution in the background biological particles. The confidence interval was initially set to 80%, corresponding to a value of 1.28, which was experimentally reasonable. The confidence interval can be modified in later experiments. The RT-HBMS can be divided into low-sensitivity, basic-sensitivity, and high-sensitivity detector modes, each with a different final alarm criterion, allowing them to be applied in various environments. The 280 nm light source used in the detector in this study can detect viruses through the RT-HBMS because it stimulates substances that comprise proteins, such as tyrosine and tryptophan. Therefore, when M13 aerosols occurred, the alarm rate was expected to be 100% for micrometer size. However, because this RT-HBMS has a minimum occurrence condition according to the background environment to reduce the false detection rate, the lowest alarm rate occurred when a clean environment was maintained before and after the occurrence. When particles were generated under the corresponding conditions, the basic sensitivity was 68%, the high sensitivity was 72%, and the low sensitivity was 66%. Therefore, it can be concluded that virus aerosols can be successfully measured using the developed small fluorescent measurement instrument. 

## 4. Conclusions

This study successfully generated narrowly dispersed virus aerosol particles and confirmed the practical feasibility of a real-time handheld bioaerosol monitoring system for virus detection. When virus particles were sprayed, the alarm rate was over 66%, even at basic, low, and high sensitivities in a clean environment with the lowest detection rate. In addition, virus particles were generated by dividing them into sizes of 1, 2, 4, 5, and 7 µm in a narrow distribution. When the generated particles were confirmed by SEM and APS, the size difference was within 3%. Our findings can enhance the accuracy and reliability of detection and monitoring systems to analyze biological warfare agents and their sources. The primary goal of monitoring and research in this area is to detect and prevent biological weapon threats, including those related to viruses. Early detection and preparedness are essential to minimize the impact of such threats, whether they are natural outbreaks or intentional acts of bioterrorism. We validated the performance of the newly developed real-time handheld bioaerosol monitoring system by detecting viruses in increasing demand, and data on the detectability of bioaerosols were acquired.

## Data Availability

Data available on request due to restrictions eg privacy or ethical. The data presented in this study are available on request from the corresponding author. The data are not publicly available due to the data provided by the Agency for Defense Development is confidential and the data must be requested after disclosing the purpose of use.

## Supplementary Materials

The following supporting information can be downloaded at: https://www.mdpi.com/article/10.3390/bios14010027/s1.
