# Supplementary material for: Detection of Micrometer-Sized Virus Aerosols by Using a Real-Time Bioaerosol Monitoring System
Source: Biosensors (Basel). 2024 Jan 2;14(1):27. doi: 10.3390/bios14010027 (PMC10813430; doi:10.3390/bios14010027)
Supplement: Supplementary file 1 [file biosensors-14-00027-s001.zip › biosensors-2553655-supplementary.pdf]

Detection of micrometer-sized virus aerosols by using a real-time bioaerosol monitoring system

Hyunsoo Seo[1], Young-Su Jeong[1,*], Jaekyung Bae[1], Kibong Choi[1], Moon-Hyeong Seo[2]

[*] Corresponding Author Dr. Young-Su Jeong

E-mail: yiyap@add.re.kr

Supporting Information

	Ratio of fluorescence (-)		
Paritcle diameter (μm)	Small	Middle	Large
1	0.78	0.51	0.00
3	0.76	0.95	0.93
4	0.78	0.94	0.91
5	0.17	0.46	0.90
7	0.07	0.31	0.91

Table S1. Table for the measured fluorescence intensity units (FIU)
